# The relationship between social adversity, micro-RNA expression and post-traumatic stress in a prospective, community-based cohort.

**DOI:** 10.21203/rs.3.rs-5867503/v1

**Published:** 2025-03-17

**Authors:** Chengqi Wang, Monica Uddin, Agaz Wani, Zachary Graham, Andrew Ratanatharathorn, Allison Aiello, Karestan Koenen, Mackenzie Maggio, Derek Wildman

**Affiliations:** University of South Florida; University of South Florida; University of South Florida; University of South Florida; Columbia University; University of North Carolina at Chapel Hill; Broad Institute of MIT and Harvard; University of South Florida; University of South Florida

## Abstract

Epigenetics influence and are influenced by the impact of social and environmental challenges on biological outcomes. Therefore, pinpointing epigenetic factors associated with social adversity and traumatic stress enables understanding of the mechanisms underlying vulnerability and resilience. We hypothesized that micro-RNAs (miRNAs) expression may be associated with post-traumatic stress disorder symptom severity (i.e., PTSS) following exposure to social adversity. To test this hypothesis, we leveraged blood-derived RNA samples (n=632) and social adversity data from 483 unique participants in the Detroit Neighborhood Health Study, a community-based, prospective cohort of predominantly African Americans. Results identified 86 miRNAs that are associated with social adversities (financial difficulties, perceived discrimination, cumulative trauma) and PTSS. These miRNAs are primarily involved in the immune response, brain and neural function, as well as cell cycle and differentiation, and 22(25%) have previously been associated with conditions related to PTSD, including traumatic brain injury and stress response. Our findings offer a fresh perspective on understanding the epigenetic role of miRNA in the interaction between social adversity and traumatic stress.

## Introduction

Post-Traumatic Stress Disorder (PTSD) is unique amongst diagnosed mental health conditions because diagnostic criteria require one or more discrete external events–i.e., trauma exposure)--for its emergence. This environmentally induced etiology indicates that the emergence of PTSD can be mediated by environmental factors, promoting an interaction between genes and the environment. Epigenetic mechanisms are one way environmental exposures can be embedded into the biology of organisms [1]. Epigenetic factors exert their influence by altering the activity of genes via activation and suppression of expression [2]. Environmentally inducible epigenetic mechanisms include DNA methylation, histone modification, and the actions of non-coding RNA including micro-RNA (miRNA) [3]. A microRNA (miRNA) is a type of small non-coding RNA molecule that plays an important role in the regulation of gene expression [4–7]. These transcripts are small (~22bp) and act to disrupt gene expression post-transcriptionally by binding to complementary sequences in mRNA transcripts. This binding primarily silences of gene expression. miRNAs are thus potent epigenetic regulators, and have been implicated in a wide range of diseases and developmental pathways [8]. The role of miRNA has been examined in a variety of mental disorders including schizophrenia, depression and PTSD [9]. While cross-sectional studies have shown that miRNAs influence risk for PTSD [10–13], less is known about how social context-related miRNA variation prospectively influences risk for PTSD or traumatic stress.

A large body of literature has established links between exposure to social adversity and adverse mental and physical health [14]. A key feature in this literature is the premise that individual-level differences in exposure to different situations and/or stimuli, combined with individual differences in responses to these stimuli, produce population-level differences in the toll exacted by these exposures [15, 16]. For example, increased risk for adverse health outcomes associated with low socioeconomic position (SEP) are hypothesized to result from greater exposure to psychosocial factors such as financial and/or occupational stressors, discrimination, and greater exposure to crime [16]. Importantly, many of these exposures have been shown to impact social and behavioral biology, including loneliness [17], discrimination [18], and stressful life event (SLE) exposure [19].

Differences in SLE exposure account for a large proportion of variation in risk for stress-related psychopathology [20, 21]including PTSD [22, 23]. This pattern is supported by findings in the Detroit Neighborhood Health Study (DNHS) cohort–the focus of the present study–where study individuals of low SEP have been exposed to a greater number of SLEs, and that this greater burden of exposure to acute and chronic SLEs, such as unemployment, difficulty accessing healthcare, and other financial problems, explains a substantial portion of the relationship between SEP and PTSD in the Detroit population [24]. However, in this prior work, even after controlling for SLEs, persons with low SEP still showed greater mean post-traumatic stress levels than those with high SEP, suggesting there are additional sources of vulnerability to stress-related psychopathology that remain to be clarified.

To address this gap in knowledge, we sought to observe how miRNA, a particular epigenetic class of molecule, reacts during the the development of traumatic stress–in particular, post-traumatic symptom severity (PTSS). Previous research suggests that some specific miRNAs show differential expression in individuals with PTSD when compared to both control and resilient subjects [25]. Indeed, these same miRNAs have been identified as components of a co-expression network closely associated with both trauma exposure and PTSD symptoms [1]. However, PTSD is a categorical variable (i.e. present or absent at a given timepoint) while symptom severity represents a continuous measure. We reasoned that these and other miRNAs could potentially be described as modifiers in influencing the connection between exposure to social adversities and PTSS. To test this viewpoint, we examined PTSS data from the DNHS, a prospective population-based longitudinal cohort based in Detroit, Michigan [2, 3]. The DNHS includes extensive data on social adversities collected in five annual waves with from 2008 to 2013, along with biospecimens collected in a subset of participants in four of the five sampling waves. This dataset is appropriate for examining the longitudinal epigenetic effects on PTSS in individuals who have experienced trauma and social adversity.

## Results

### Small RNA sequencing was conducted on samples from the DNHS in waves two and four.

To investigate the influence of miRNA in modifying the connection between social adversity and PTSS, we conducted small RNA sequencing of blood from 483 human subjects. Our dataset includes 632 individual blood samples, comprising 389 samples from wave two and 243 from wave four. 185 participants were sampled in both waves. Following quality control, adaptor trimming, and merging of paired-end reads, approximately 10 billion merged reads were mapped back to annotated mature miRNAs, averaging ~14.7 million reads per sample (**Supplemental Fig. 1**). To assess the reproducibility of our sequencing results, each sequencing plate included Qiagen Human XpressRef Universal Total RNA as a positive control. The mean Pearson correlation among these control samples was 0.98, indicating a high level of reproducibility in our sequencing data. The miRNA with the most read counts identified was hsa-mir-16, with eight miRNAs from the hsa-let-7 family nearly as abundant. The hsa-let-7 family is known for its significant roles in gene expression regulation and involvement in various physiological activities including metabolic regulation, cell differentiation and proliferation [26]. Other dominant miRNAs quantified by total read counts across all samples, such as hsa-miR-486-5p, have previously been demonstrated to be abundant in red blood cells, further highlighting their prevalence in the present context [27].

The sequencing results from two distinct waves of sampling in multiple years offer a unique opportunity to explore the longitudinal influence of miRNA on the association between social adversity and PTSS: (i) the miRNA profiles from wave two can be examined to understand the prospective relationship between PTSS in wave three and social adversity reported in wave two and three; and (ii) the miRNAs from wave four can be utilized to study the relationship between PTSS in wave four and social adversity reported in wave three and four. We opted to focus on wave four rather than wave five, because the latter sampled a smaller number of DNHS participants.

### PTSS can be estimated by lifetime social adversities

We endeavored to identify the most suitable distribution for estimating PTSS, ensuring the selection of an appropriate general linear regression model (GLM). To do so we utilized maximum log-likelihood estimation to optimize the parameters and employed the Bayesian Information Criterion (BIC) [28] to evaluate four frequently used distributions: negative binomial (NB), Poisson, Gamma, and Weibull. The Gamma and NB distributions provide the best fit for wave three and four PTSS, as evidenced by their lowest Bayesian information criterion (BIC) profiles (**Supplemental Table 1, Supplemental Figs. 2,3**). The 1,000 bootstrapping replicates demonstrate similar performance of Gamma and NB on PTSS profile fitting (p=0.575 and p=0.615 for BIC of NB fit in wave three and four respectively, **Supplemental Fig. 4**). We opted for NB due to its compatibility with the categorical nature of PTSS variables, (as shown in **Supplemental Fig. 2B, 3**) and its widespread use in modeling survey data [29–31].

We employed NB regression for the testing of key factors contributing to PTSS. These factors include lifetime and current social adversity profiles for key factors identified as important to prospective PTSS in our prior work [32] (i.e. cumulative trauma, loneliness, perceived discrimination, financial difficulties, and emotional mistreatment). Additionally, we investigated the role of genetic background of individuals, quantified as a polygenic risk score (PRS), and cell surrogate proportion [33], in contributing to PTSS in the context of all adversity factors. We utilized adversity data from either wave two or a combination of waves two and three to predict PTSS in wave three, while wave four PTSS was predicted using adversity factors from wave four (**Supplemental Tables 2–4**. See methods for detail). Our findings from the modeling of wave four PTSS suggest comparable model performance when using solely lifetime social adversity profiles in contrast to incorporating both lifetime and current profiles, or along with PRS (10-fold cross-validation Spearman correlation of 0.624 vs. 0.623, **Supplemental Table 4**). A similar pattern emerged in the prediction of PTSS for wave three, where using both wave two and three social adversity profiles resulted in 10-fold cross-validation Spearman correlations of 0.632 for solely using lifetime profiles and 0.639 for the combination of both lifetime and current profiles (**Supplemental Table 3**). These results also suggest a limited contribution of emotional mistreatment to PTSS prediction when considering only lifetime social adversities. (Wald test p > 0.5, **Supplemental Tables 3 and 4**). An exception occurred when exclusively utilizing wave two social adversity profiles for predicting wave three PTSS, where there was approximately a 0.05 decrease in the Spearman correlation coefficient when using solely lifetime profiles compared to using a combination of lifetime and current profiles (**Supplemental Table 2**). The incorporation of PRS or cell surrogate proportion led to a reduction of Spearman correlation, typically ranging 0.01 to 0.05 in most instances compared to utilizing lifetime social adversity profiles (**Supplemental Tables2–4**). Our findings indicate that NB regression using only lifetime social adversity yields comparable model performance to including both current and lifetime profiles. Given the larger sample size accessible for lifetime social adversity (Wave three prediction, **Supplemental Tables 2, 3**) and the reduced model feature space, we opted to include only lifetime profiles for our subsequent analyses. This model omits the inclusion of PRS and cell surrogate, given their limited contribution to PTSS prediction (**Supplemental Table 4**).

Several earlier studies have shown that prior PTSS has the highest predictive capability for predicting the future risk of PTSS [32, 34–37]. Here, we also sought to integrate past PTSS data into our NB regression models for forecasting PTSS in waves three and four. We found strong Spearman correlation coefficients for our predictions. Wave three PTSS predicted from wave two social adversity factors determined a Spearman correlation of 0.962 in 10-fold cross-validation (detailed data not shown). Similarly, the combination of wave two and three factors led to a correlation of 0.958 for wave three predictions, and the combination of wave four and three factors yielded a correlation of 0.94 for wave four predictions (detailed data not shown). These findings validate our earlier observations that prior post-traumatic psychopathology ranked highest in predicting the prospective risk PTSD [32]. However, it’s important to note that these levels were largely influenced by the substantial correlation between PTSS measurements in different waves, as illustrated in **Supplemental Table 5**, where correlations exceeded 0.86. Additionally, given our focus on exploring the link between social adversity and PTSS, incorporating PTSS data from previous waves might restrict the impact of other factors on PTSS prediction. For instance, our established NB regression demonstrated the significant contribution of lifetime social adversity factors to wave four PTSS (as indicated by the F test, p < 0.05, **Supplemental Table 4**). However, when PTSS from prior waves were incorporated into the model, the contribution of these factors diminished (data not shown). Notably, we observed significant contributions only from trauma and wave three PTSS to wave four PTSS. Besides the prior wave PTSS profile, the stronger association between lifetime social adversity profiles and PTSS compared to other factors leads us to use lifetime social adversity to model PTSS.

### Identifying miRNA signatures that modify the relation between social adversity and PTSS.

**Figure 1:**
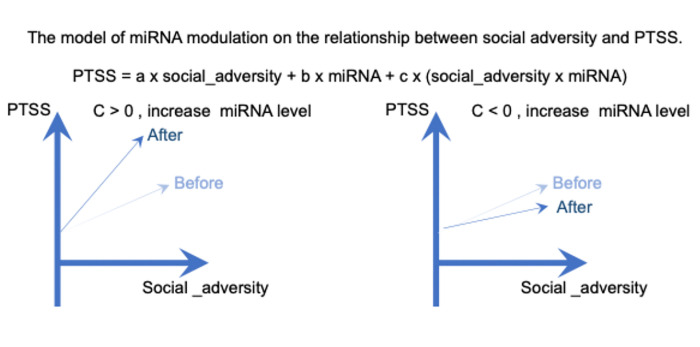
The interaction test within a general linear regression is employed to detect miRNA linked to the impact of social adversity on PTSS. The coefficient *c* signifies the interaction effect between social adversity and miRNA expression concerning PTSS outcomes. The graphs illustrate the association slopes between social adversity and PTSS, with light and dark blue denoting slopes before and after elevated miRNA expression. In the left plot, heightened miRNA levels are associated with an increase in PTSS in the context of a particular social adversity exposure, whereas the right plot suggests the converse effect, with increased miRNA showing a potentially protective effect against PTSS in the context of a particular social adversity exposure.

**Figure 2:**
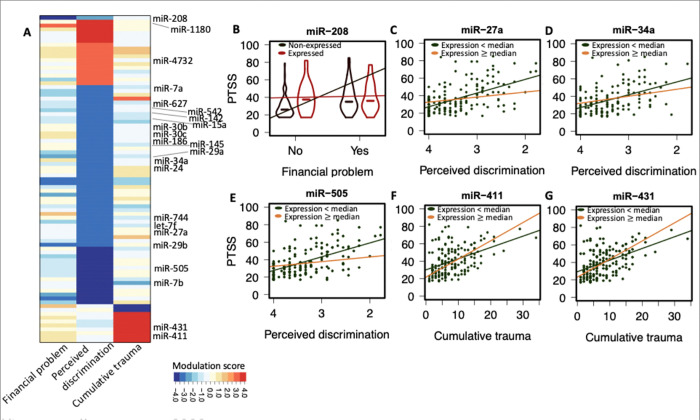
The statistical framework reveals the influence of miRNA signatures on the impact of social adversity on PTSS. **A**. miRNAs play a significant moderating role in the associations between social adversity and PTSS, as determined by a two-sided Wald test with a false discovery rate (FDR) threshold of 0.1. The modulation scores of miRNAs are presented, defined as the z score, obtained by dividing the coefficient (c) by the standard error in NB regression. The 22 highlighted miRNAs are those associated with previously reported signatures linked to PTSD-related phenotypes. **B**. The expression of miR-208 is associated with the connection between financial difficulties and PTSS. This relationship is illustrated in the scatter plot. Samples are categorized based on their miR-208 expression profiles. Among participant samples lacking miR-208 expression, there is a positive correlation between financial problems and PTSS. However, for samples expressing miR-208, this correlation diminishes. **C-E**. The relationship between PTSS and perceived discrimination is depicted. A lower value on the horizontal axis indicates that participant perceived higher levels of discrimination. The red and blue samples specifically represent participant samples with high and low miRNA expression base on the median profile. The association between perceived discrimination becomes more pronounced when miRNA levels are lower. **F-G**. Similar to C-E, it illustrates the relationship between PTSS and cumulative trauma. The association between cumulative trauma and PTSS intensifies as miRNA levels increase.

NB regression was used to assess the impact of miRNAs on PTSS, incorporating all five previously mentioned social adversities. A total of 323 individuals from wave three and 243 individuals from wave four with social adversity profiles and miRNA expression data, were included in this analysis (Table 1 and **Supplemental Fig. 5**). PTSS was represented by the sum of miRNA expression levels and lifetime social adversities (individual variables of cumulative trauma, loneliness, perceived discrimination, financial difficulties, and emotional mistreatment, see Methods for details) within the NB regression framework. The Wald test [28]was employed to examine the direct contribution of miRNA expression to PTSS. We identified four miRNAs contributing to PTSS in wave three (**Supplemental table 6**), and zero miRNAs contributed to PTSS in wave four. We further conducted research to identify whether miRNAs might modify the relationship between social adversity and PTSS. Two variables exhibit interaction when the impact of one variable is contingent upon the presence or values of the other variable [38]. In our case, the effect of social adversity or the association with PTSS could depend on miRNA abundance, which is the typical case of interaction between variables. The interaction of any two variables related to PTSS profiles can be tested by NB regression (Fig. 1). We included all five lifetime social adversity measures in the NB regression, aligning with the model for exploring the main effect of miRNAs. The coefficient c of the multiplication term indicates the level of the interaction effect, and the Wald test can evaluate its statistical significance [39]. We calculated a score for each lifetime social adversity profile, illustrating the influence of each miRNA interaction with a particular social adversity on PTSS in either wave three or four (Methods), where the z score of each miRNA’s interaction is the interaction coefficient c divided by its standard error. We identified 74 miRNAs that significantly modify the association between perceived discrimination and PTSS in wave three, one miRNA that modifies the relationship between lifetime financial difficulties and PTSS in wave three, and 10 miRNAs that modify the association between lifetime cumulative trauma and PTSS in wave four (False Discovery Rate, FDR < 0.1, Fig. 2A, **Supplemental Table 7–9**). The results include 22 miRNAs that have been previously reported in association with conditions or traits related to PTSD [11, 13, 40–53], including traumatic brain injury and stress response (**Supplemental Table 10**).

Our findings provide new insight into of a group of miRNAs previously identified in a co-expression network of PTSD [13]. The linear combination of these miRNA concentrations in the originally reported cluster showed significant differences between individuals with PTSD and resilient individuals, as well as trauma-exposed individuals to nonexposed controls. However, none of these miRNAs individually showed differential expression in this study [13]. Our findings provide evidence that some of the actions of these miRNAs may influence PTSD symptoms by in association with the effects of social adversities. For example, the PTSS profiles from wave two to wave three in the individuals expressing miR-208 are less associated with financial problems, as evidenced by the shallower slope of the red line in Fig. 2B. Conversely, participants with higher expression levels of miR-27a, miR-34a, and miR-505, may experience a protective effect against PTSS in the context of the investigated social adversities. In contrast, participants with higher expression levels of miR-411 show the opposite trend, with their PTSS being worsened in the context of the investigated social adversities (Fig. 2C–F). An additional instance of our model’s application is demonstrated with miR-431. Previous findings from a mouse model indicated that miR-431 has the potential to heighten the susceptibility of the inner ear to noise-induced trauma [50]. Our findings also suggest that the augmentation of miR-431 results worsen PTSS in response to cumulative trauma (Fig. 2G).

### The identified miRNA signatures are involved in immune response, cell cycle and differentiation, as well as brain and neural functions

**Figure 3:**
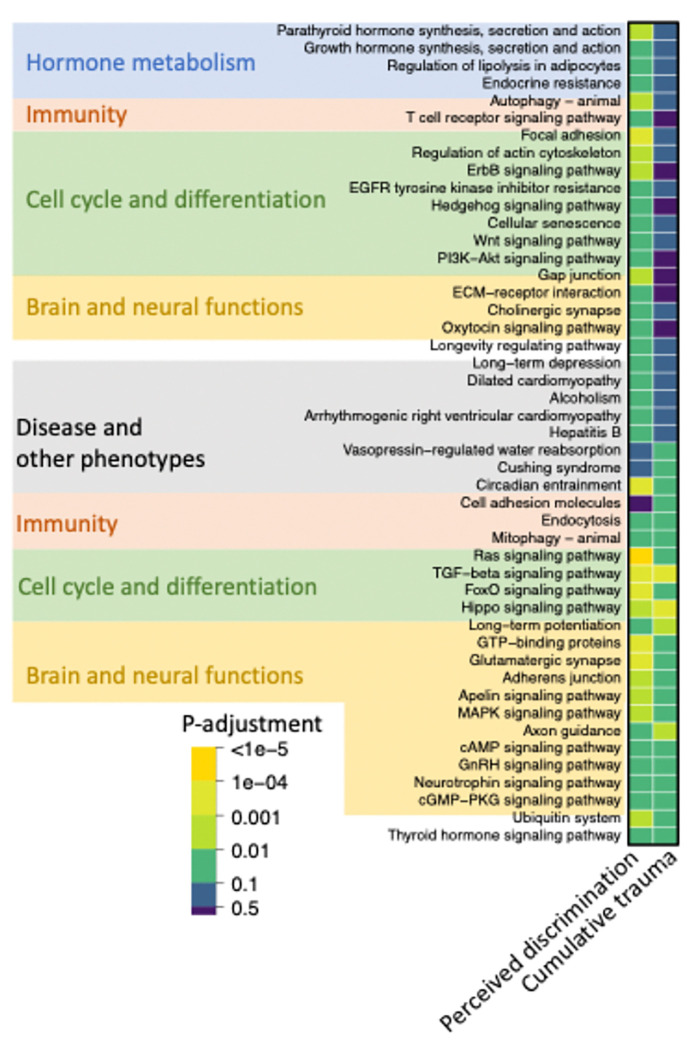
KEGG pathway enrichment analysis was conducted to explore miRNAs that could potentially modulate the association between PTSS and perceived discrimination, or between PTSS and cumulative trauma. Each pixel reflects the enrichment level.

Our next goal was to identify the biological pathways targeted by the miRNAs we identified as potentially involved in the modulating relationship between social adversity and PTSS (i.e. 74 miRNAs for perceived discrimination, 10 miRNAs for cumulative trauma and one miRNA for financial difficulties). To this end, we downloaded annotated target genes for each miRNA identified in our study from miRDB (https://mirdb.org/) with a stringent binding score cut off 80 [54]. We then performed KEGG enrichment analysis [55] for the miRNAs identified for each social adversity. This procedure identified 94 unique KEGG terms enriched in the target genes of miRNAs that modulate PTSS through interactions with social adversities (Supplemental Table 11). These terms (amongst others) are categorized into hormone metabolism, immunity, cell cycle and differentiation, brain and neural functions, and diseases (Fig. 3). We presented these categories based on whether they are involved in modulating either perceived discrimination, trauma, or both (the financial problem was excluded because only a single miRNA was found to influence the relationship between lifetime financial difficulties and PTSS).

## Discussion

The current study demonstrates a significant, prospective association between blood-based measures of miRNA and traumatic stress following exposure to social adversity in a community-based, prospective cohort of predominantly African Americans. In the context of specific social adversity experiences, individuals exhibiting distinct miRNA expression patterns may be at an elevated risk of PTSS. Specifically, we found a group of miRNAs that can either intensify or diminish the correlation between social adversity and PTSS as their expression profiles increase. Conversely, individuals lacking this miRNA expression pattern may enjoy a protective effect against PTSS, even when experiencing similar levels of social adversity. Several of these identified miRNAs have been previously linked to traumatic brain injury [51–53], underscoring and strengthening earlier research that has demonstrated an association between traumatic brain injury and PTSD [56, 57]. Furthermore, the target genes of the identified miRNAs primarily participate in immune response, cell cycle regulation and differentiation, brain and neural functions, and other diseases related to PTSD. This offers additional evidence connecting these pathways to PTSD. Importantly, our findings emphasize a fresh viewpoint on these pathways implicated in PTSD, potentially affecting the interaction between social adversity and PTSS. Our study contributes to the understanding of how social adversity influences psychological outcomes, driven by underlying biological molecular mechanisms.

Our analysis indicates that lifetime social adversity has a similar predictive capacity compared to the linear combination of all current social adversity factors. Lifetime social adversity, which aggregates the average profiles across all considered waves, also ensures a higher sample size, as some individuals missed specific adversity surveys in certain waves (**Supplemental Tables 3, 4**). Within our statistical framework, lifetime social adversities were employed to investigate whether miRNA modified PTSS following exposure to social adversity. Perceived discrimination and loneliness data are only available for wave three. The wave three PTSS survey enabled us to explore potential miRNA contributions, measured in wave two, to PTSS outcomes following exposure to perceived discrimination and loneliness reported in wave three. While we were only positioned to identify miRNAs in wave two that significantly modified the association between perceived discrimination and PTSS in wave three, we still observed that these miRNAs exhibited significantly higher modulation scores in wave four compared to other miRNAs (**Supplemental Fig. 6A**, Wilcoxon test < 1e-3). This observation partially validates our study framework, demonstrating that miRNAs identified in one wave retain or exhibit consistent effects in subsequent waves. Similarly, the identified miRNAs in wave four, significantly modifying the association between lifetime cumulative trauma and PTSS, also demonstrated significant modulation score compared with other miRNAs in wave two (**Supplemental Fig. 6B**, Wilcoxon test < 1e-3). These findings underscore the robustness of our study design in identifying modifying miRNAs.

Upon ordering all KEGG terms based on their enrichment p-adjustment values (Fig. 3, **Supplemental Table 11**), we observed a cluster of functional terms that are shared by two groups of miRNAs, which modulate perceived discrimination and cumulative trauma(The financial problem was excluded because only a single miRNA was found to influence the relationship between lifetime financial difficulties and PTSS). The Ras signaling pathway has previously been linked to PTSD [58], whereas the TGF-beta and Hippo signaling pathways have been recognized as significant connections to stress-regulated pathways [59, 60]. These pathways are intricately involved in cell growth, proliferation, and differentiation processes [61]. Moreover, the pathways identified includes long-term potentiation, glutamatergic synapses, MAPK, cAMP, neurotrophins, and cGMP-PKG, which collectively form vital components of the intricate network governing synaptic plasticity, neuronal signaling, and cognitive function in the brain [62–66]. Our findings also highlight the involvement of two immune response pathways, endocytosis and mitophagy, which have been previously associated with PTSD [67, 68]. Overall, our results offer novel insights into the pathways previously reported in PTSD, elucidating their regulation potentially via miRNA and their impact on the relationship between social adversities and PTSS.

Our research introduces a well-specified statistical framework for identifying a biological molecular signature that underlies associations between social adversity and PTSS. This framework can be applied to investigate any third factor that may potentially modify known one-to-one relationships. A similar framework has been employed to explore the human immune response over time in response to varying levels of racial discrimination [69]. While this robust framework has the potential to uncover protective biological factors that counteract adverse influences, it falls short of establishing a causal relationship between the identified miRNA and the heightened risk of PTSD. Further experimentation is necessary to validate the contribution of the identified miRNA or related biological factors. Nonetheless, our study lays the groundwork for understanding the inherent vulnerability factors influencing psychological health disparities.

We included many lifetime social adversity factors, except emotional mistreatment, which showed limited ability to predict PTSS (**Supplemental Tables 3, 4**), in our NB regression analysis to explore the interplay between social adversity and miRNA on PTSS outcomes. It is important to emphasize that the social adversity data are derived from annual survey responses, reflecting a potentially prolonged period of individual exposure to social adversity. In contrast, miRNA was extracted at a specific time point, capturing the expression profile only at that particular moment. Even though the miRNA maintains a high consistency between waves two and four, as reflected by a Spearman correlation greater than 0.75 in the majority of individuals (**Supplemental Fig. 7**), the fluctuation in miRNA expression over time could still influence their ultimate contributions to PTSS. Though sequencing multiple time points and statistically testing the consistent modulation potential of miRNA across all waves is a logical approach, as demonstrated in our studies and consistent in waves three and four (Supplementary Fig. 5), we should aim to enhance experimental design and statistical testing to simultaneously incorporate all sequencing data comprehensively. This will necessitate the inclusion of more individual samples in our study.

In general, it’s both challenging and essential to pinpoint the biological factors that influence the outcome of traumatic stress among individuals exposed to social adversities. To address this, we have introduced a robust statistical framework to comprehend how miRNA, an understudied epigenetic component in both community-based contexts and PTSD, regulates the connection between social adversity and PTSS. Several miRNAs identified here target genes involved in pathways previously associated with PTSD, suggesting that genomic variation in this understudied element can help improve our understanding of vulnerability to PTSD in the face of exposure to distinct social adversities. Overall, our discoveries provide new insight into how miRNA shapes the interaction between social adversities and PTSS.

## Methods

### Detroit Neighborhood Health Study (DNHS)

In this investigation, we utilized data from the Detroit Neighborhood Health Study (DNHS), a prospective population-based longitudinal cohort consisting of individuals residing in Detroit, Michigan [70, 71]. All participants involved in this study were aged 18 years or older and predominantly self-identified as African American (AA). The primary objective of the DNHS was to examine how biological variation, stressful and traumatic life experiences, and environmental factors contribute to the prediction of psychopathology and behavior. Participants underwent structured telephone interviews annually between 2008 and 2013 to assess various aspects, including perceptions of their neighborhoods, mental and physical health status, social support, exposure to traumatic events, symptoms of post-traumatic stress disorder, depression, and generalized anxiety, as well as alcohol and tobacco use. Informed consent was obtained at the outset of each interview and reconfirmed at the time of specimen collection. Further details regarding DNA and RNA isolation procedures are provided in [72, 73], respectively. The Institutional Review Boards at the University of Michigan and University of North Carolina reviewed and approved this study.

### Polygenic risk score (PRS)

A polygenic risk score (PRS) for PTSD was calculated for each participant using summary statistics from the most recent genome wide association study (GWAS) of individuals with African ancestry from the Psychiatrics Genomics Consortiums PTSD Working Group excluding participants from the DNHS [74]. For each participant, PRS for PTSD were calculated by taking the weighted sum of risk alleles, with each allele weighted by the z-scores from the GWAS summary statistics using PRSice-2 [75, 76]. Risk alleles for PTSD were selected using p-value thresholds ranging from 5 × 10^−8^ to 1 with the best fitting PRS determined by the maximum variance in DNHS PTSD diagnosis explained based on Nagelkerke’s R.

### Measures

In this study, exposure to traumatic events throughout life was evaluated using a survey consisting of 19 items, consistent with previous research [71]. In addition, we examined exposure to five measures of social adversity implicated in PTSS based on earlier work [77]. These included: perceived discrimination, assessed using the Everyday Discrimination Scale (EDS), a nine-item self-report questionnaire [78]; loneliness, gauged using a standard three-item scale [79]; and emotional mistreatment and financial problems, which were evaluated as independent stressors [70, 80]. Our outcome measure was a continuous measure of PTSD severity, calculated by summing scores for 17 symptoms (i.e. PCL-C) [81] stemming from the most severe lifetime trauma experienced by each participant. Participants self-reported demographic information including age, sex, race, education, marital status, and employment status. The current social adversity was characterized by either the accumulation of adverse social events (cumulative trauma) or the experience of any social adversity events (emotional mistreatment and financial problems) since the last interview in the preceding survey wave. Perceived discrimination was assessed by aggregating the responses across nine individual survey items in the EDS included in wave three [78], while loneliness was quantified by combining the results from three individual survey items in the loneliness scale, included in wave three. Lifetime trauma was determined by totaling the cumulative trauma exposure reported across all previous sampling waves. Lifetime emotional mistreatment and financial problems were assessed based on whether any emotional mistreatment or financial issues were experienced, considering all previous waves.

### Small RNA sequencing

The quality and quantity of the total RNA samples are assessed using Qubit fluorometric concentration (Invitrogen, Waltham, MA), and Tapestation electrophoresis integrity (Agilent, Santa Clara, CA). This ensured that only high-quality RNA was used for subsequent library preparation. In total 638 samples (389 from wave 2 and 243 from wave 4) were selected originating from 483 unique participants. Library preparation was performed following the QIAseq miRNA Prep Kit protocol (QIAGEN, Hilden, Germany) with QIAseq miRNA unique dual indices (Set A-H; Qiagen). Final cDNA libraries were assessed for quality via Qubit (Invitrogen, Waltham, MA) and TapeStation (Agilent, Santa Clara, CA). miRNA sequencing was performed on the NextSeq 2000 platform (Illumina, San Diego, CA) using the P3 reagents/flow cell with 66bp single read and 10bp dual indexing (Illumina).

### Small RNA mapping

The quality control of sequencing results was performed by FASTQC [82] and MULTIQC [83]. The sequencing adaptor is removed by the Trim Galore [84]. The forward and reverse reads were merged by using the PEAR [85]. The mirDeep2 [86] was implemented to map the merged reads back human genome GRCh38 (hg38). The precursor and mature miRNA information was downloaded from miRbase [87].

### Cell surrogate proportion

The cell surrogate proportion was calculated by CellCODE [33] to estimate the relative composition of BCell, CD8T, CD4T, NKCell, Mono and Gran cells.

### Statistical modeling

We evaluated four standard probability distributions to fit PTSS: 1) Poisson, 2) Gamma, 3) Negative Binomial (NB) and 4) Weibull. Maximum log-likelihood estimation was used to optimize the parameters and we employed the Bayesian Information Criterion (BIC) to rank the model fitting performance. NB was chosen as the optimal model for our data. We then used Spearman correlation from 10-fold cross-validation in NB regression to estimate the contributions of different factors, including gender, social adversities, PRS and cell surrogate proportion to PTSS prediction. In the 10-fold cross-validation process, the dataset was partitioned into ten roughly equal-sized subsets, referred to as folds. Our linear model was trained using nine of these folds, reserving one-fold for testing the model’s performance. This procedure was repeated ten times, ensuring that each of the ten folds served once as the test set. The performance of the model was averaged across all ten iterations to derive an estimation of its performance. The 10-fold cross-validation in NB regression with L1 penalty was also utilized to validate the results.

An interaction test within NB regression was utilized to pinpoint miRNAs that might influence the interplay between social adversities and PTSS. In statistical terms, two variables interact when the impact of one variable is dependent upon the state of the other [38]. A multiplication term in a multivariate linear model can assess this interaction effect between two variables. We constructed a linear model $PTSS=a_{j}\times social\_{adversity}_{j}+b\times miRNA+c\times (social\_{adversity}_{j}\times miRNA)+\sum_{i\neq \;j}a_{i}\times social\_{adversity}_{i}$, where “miRNA” denotes the expression level of a specific miRNA under examination. The model enables a direct investigation of the contribution of a specific social adversity $a_{j}\times {social\_adversity}_{j}$ and a specific miRNA b×miRNA, as well as the interaction between social adversities and miRNA $c\times \left(\left.{social\_adversity}_{j}\times miRNA\right)\right.$ to PTSS. Additionally, we controlled for the contributions of other social adversities, represented as $\sum_{\;i\neq j}a_{i}\times {social\_adversity}_{i}$. The primary contribution of each miRNA to PTSS was indicated as b×miRNA, where b represents the linear coefficient. The relationship between the investigated social adversity and PTSS can be described as $a_{j}+c\times miRNA$. The estimate (c) represents the modulatory effects of miRNA on PTSS. If the coefficient c is positive, it implies that higher miRNA expression levels are associated with increased PTSS in the context of a given social adversity (positive effect). Conversely, if c is negative, higher miRNA levels are associated with reduced PTSS in the context of a given social adversity, suggesting a protective effect. The modulation score for each miRNA is defined as the Wald test z score, calculated as the coefficient c divided by its standard error. To identify significant miRNAs in the interaction test, we employed false discovery rates (FDRs) to adjust the two-sided Wald test P-values, selecting miRNAs with an FDR below 0.1 as significant candidates in the interaction test.

The main effects of miRNA were modeled using the previously described linear negative binomial (NB) framework without interaction terms. Specifically, the linear model can be expressed as $PTSS=\sum_{i}a_{i}\times {social\_adversity}_{i}+b\times miRNA$. The Wald test was then applied to evaluate whether the expression of the specific miRNA significantly contributes to PTSS.

#### KEGG enrichment analysis

To elucidate the biological pathways influenced by miRNAs, we retrieved all annotated target genes for each miRNA identified in our study from the miRDB database (https://mirdb.org/) using a stringent binding score threshold of 80 [54]. To assess the functional relevance of these miRNAs, we performed pathway enrichment analysis to determine whether specific KEGG pathways were significantly enriched among the target genes. Our focus was on miRNAs identified in the modulation of the relationship between social adversities and PTSS. The enrichment analysis was conducted using a hypergeometric test with p-values adjusted by the False Discovery Rate (FDR), and only pathways with an adjusted p-value < 0.1 were considered significantly enriched.

## Figures and Tables

**Table 1.** Demographic characteristics of the individuals involved in the statistical framework for studying the modulatory effects of miRNA signatures on the relationship between social adversity and PTSS.

Demographic Profile	Wave Two	Wave Three	Wave Four
Age, mean(SD)	56.5(15.5)	57.4 (15.6)	59.8 (14.1)
Gender, Female(%)	187 (57.8)		140 (57.6)
Self-reported Race, Black or African American (%)	256 (79.5)		196 (80.7)
Self-reported Race, American Indian or Alaska Native (%)	3 (0.9)		3(1.2)
Self-reported Race, Native Hawaiian or Other Pacific Islander (%)	1 (0.3)		1 (0.4)
Self-reported Race, White (%)	46 (14.2)		35 (14.4)
Self-reported Race, other (includes those who only specified Hispanic for this question and mixed races) (%)		16 (5.0)	8 (3.3)
Lifetime trauma events, mean (SD)	5.8 (3.9)		7.9 (5.3)
Lifetime financial problems, Yes (%)	202 (62.5)		165 (67.9)
Lifetime emotionally mistreated, Yes (%)	102 (31.6)		95 (39.1)
Loneliness scale, mean (SD)		1.4 (0.5)	1.4 (0.6)
Perceived discrimination, mean (SD)		3.4 (0.5)	3.3 (0.6)
PTSS, mean (SD)		36.5 (15.4)	39.6 (16.2)
